# The Impact of Heat Load on Behaviour and Physiology of Beef Cattle: Preliminary Validation of Non-Invasive Diagnostic Indicators

**DOI:** 10.3390/ani16020308

**Published:** 2026-01-19

**Authors:** Musadiq Idris, Megan Sullivan, John B. Gaughan, Clive J. C. Phillips

**Affiliations:** 1 Faculty of Veterinary and Animal Sciences, The Islamia University of Bahawalpur, Bahawalpur 63100, Pakistan; 2 School of Agriculture and Food Sciences, Gatton Campus, The University of Queensland, Gatton, QLD 4343, Australia; megan.sullivan12@outlook.com (M.S.); j.gaughan@uq.edu.au (J.B.G.); 3 Curtin University Sustainability Policy (CUSP) Institute, Curtin University, Perth, WA 6845, Australia; clive.phillips@curtin.edu.au; 4 Institute of Veterinary Medicine and Animal Sciences, Estonian University of Life Sciences, Kreutzwalki 1, 51014 Tartu, Estonia

**Keywords:** behaviour, beef cattle, heat load, infrared thermography, physiology

## Abstract

Early diagnosis of heat load in beef cattle remains a challenge due to the limited understanding of behaviour-based indicators. This preliminary longitudinal study aimed to validate behavioural and physiological responses previously identified as heat load indicators. Black Angus steers were exposed to high environmental temperatures expected to cause heat load in the following sequence: an initial thermoneutral period, a hot period, and a recovery period. Changes in the positioning of key body parts, feeding behaviour, body maintenance, respiratory dynamics, and eye temperature were monitored. In the hot period, cattle increased their respiration rate, panting, and infrared eye temperature. Increased stepping by their left limbs suggested involvement of the right brain hemisphere in a stress response to high environmental temperatures. Cattle also held their heads more downward, ears backward, and their tail vertical, and reduced eating, grooming, and scratching during the hot period. Cattle responses to hot conditions were persistent in the recovery period, reflecting diagnostic relevance of the head, ear, and tail movements, stepping, especially by left limbs, and infrared eye temperature as non-invasive tools to identify heat load condition in cattle. The study reinforces our understanding of the specific behavioural and physiological responses to heat load condition, especially those involving left-limb stepping, ear and tail posture, and infrared eye temperature, are reliable indicators for identifying cattle experiencing high environmental temperature.

## Simple Summary

Identifying heat load in feedlot cattle can be difficult, with the high density of cattle obscuring behavioural signs that might indicate the problem to workers. This study investigated changes in subtle behavioural cues such as lateralised limb movement, ear and tail positioning, and infrared eye temperature that could serve as early, non-invasive indicators of heat load in beef cattle. By confirming these indicators under controlled heat load conditions and distinguishing responses indicating heat load, the research offers a novel approach for preliminary validation of non-invasive diagnostic indicators for a precision livestock stress monitoring in high environmental temperatures.

## 1. Introduction

High environmental temperatures are a significant threat to the tropical and subtropical beef farming industries due to economic losses arising from the impact of heat load on health and production, as well as mortality of beef cattle [1]. High environmental temperatures provoke both behavioural and physiological responses of cattle to maintain and restore thermal balance as a coping strategy [2,3,4]. Thermal acclimation is critical for production and differs between beef breeds, reported as nine days for Angus and fourteen days for Charolais [5]. Thermal tolerance is considered an adaptive response of cattle [6]; however, thermo-tolerant animals have lower production potential, with the ensuing reduction in feed intake greatly reducing growth rates.

Under high environmental temperatures that lead to heat load, cattle adapt their behaviour by increasing water consumption, standing time, respiration, and panting rates, accompanied by a decline in feed intake [7,8]. The cattle seek shade in an attempt to reduce the radiant heat load from the sun [9]. Evaporative cooling is the major source of heat loss from the body, especially during inspiratory/expiratory exchanges. Increased respiration rate (RR) and panting score (PS) are early signs of increasing heat loss from the body [10], which may coincide with an increase in water consumption. In one study, Black Angus steers drank 41 ± 0.96 L/d during heat load, compared with cattle in a thermoneutral environment, which drank 30 ± 0.85 L/d [11]. Under heat load, cattle may reduce feed intake, which serves to diminish heat output from feed digestion [12]. They prefer to stand rather than lie down to increase the body surfaces available for heat loss by sweating and evaporation. Other behavioural changes include crowding over water troughs, reduced or no rumination, wallowing, and water splashing [13]. The adaptive nature of these behavioural responses to high environmental temperatures is still unclear. There may also be psychological elements in the responses, such as movement responses to stressors, which are neutral or even maladaptive as they increase heat production. Body part movements include stepping, ear, head, and tail movements. These body movements have been investigated in response to a variety of stressors, but not in response to a high environmental temperature [14,15,16].

Among the physiological responses to hot environmental conditions, increases in core body temperature, respiratory exchange, and sweating are widely reported in heat-stressed cattle [17]. Increased body temperature is a major indicator of heat stress in cattle. Under thermoneutral conditions, the core body temperature of cattle ranges from 38.0 to 39.3 °C [18]. During high environmental temperatures, animals increase heat dissipation from the skin through sweating, vasodilatation, and greater peripheral circulation [2]. Infrared thermography can detect these changes, especially in the eye around the *caruncula lacrimalis* [19], which is rich in blood supply. Measurement of eye temperature using infrared thermography has been a useful indicator of pain and stress in animals that are being dehorned, castrated, or are anxious [20,21,22].

Our earlier investigations were mainly focussed on dietary modulation during high environmental temperatures and compared the behaviour and physiological responses of feedlot cattle to different dietary treatments. These confirmed that the cattle suffering heat load conditions exhibit changes in specific behavioural and physiological parameters such as backward-oriented ears, downward head position, vertical tail, increased left-limb stepping, and elevated infrared eye temperature [2,4]. These responses were identified as potential non-invasive markers for early detection of heat stress.

Unlike our previous investigations that were primarily focussed on comparing different dietary modulation, the current study was designed as a preliminary validation and investigation of the diagnostic consistency of these behavioural and physiological indicators under replicated high environmental temperatures, independent of dietary comparisons. Specifically, we aimed to determine whether the observed changes, particularly in body part positioning, stepping asymmetry, and infrared eye temperature, are persistent, reproducible, and thus suitable for use as early, non-invasive indicators of heat load in high environmental temperatures. Timely identification of stressed cattle could enable prompt alleviation strategies, improving both welfare and productivity.

## 2. Materials and Methods

### 2.1. Animals and Treatments

Ethical approval for the study was obtained from the Animal Ethics Committee (SAFS/570/16) of the University of Queensland (QLD), Australia. Twelve (12) yearling Black Angus steers were procured from a commercial feedlot in the Darling Downs region of QLD, Australia, for a study at The University of Queensland’s Animal Science Precinct (27.6° S, 152.3° E). The animals had an initial non-fasted body weight of 461 ± 8.8 kg.

Briefly, the steers were moved to the experimental feedlot pens (5 d) from a commercial feedlot (25 d), and then these 12 steers were kept in individual pens before moving them to two climate-controlled rooms (CCR) for 22 d. The animals were exposed to an initial thermoneutral period (TN; d 1–7), a transition phase to hot conditions (TP1; d 8), a hot period (HOT; d 9–15), a transition phase from hot conditions (TP2; d 16), and a recovery thermoneutral (Recovery; d 17–22) period (Table 1). In the TN and Recovery periods, the ambient dry bulb temperature (T_A_) and relative humidity (RH) in the controlled rooms was maintained at 20 °C and 65%, respectively. In the HOT period, T_A_ and RH increased hourly each day from 0700 h to reach a maximum at 1100 h (Table 1), which was maintained until 1500 h and then decreased hourly from 1500 h to the daily minimum T_A_ and RH at 2000 h. The T_A_ declined over the HOT period to prepare cattle for Recovery.

### 2.2. Animal Housing and Management

The animals were kept in standard housing facilities and provided with best management practices concerning immunisation, feeding, and drinking before the start of the experiment in the CCR, as detailed previously [4]. Briefly, on entry to the experimental feedlot, steers were offered a feedlot finisher diet based on cereal grain until the end of the trial, except for during the HOT period in the climate control chamber (Table 2). Three days prior to the HOT period, the animals were fed a substituted diet (alfalfa hay for grain) in the climate-controlled rooms. They were transitioned back to the finisher diet over three days in the Recovery period. In the substituted diet, 8% of the grain was substituted by an isoenergetic amount of alfalfa hay during this period to improve heat load endurance by the cattle [4].

Cattle had refusals removed and weighed prior to the provision of 50% of the ration at 0900 h and the remainder at 1300 h. Feed dry matter content was determined by oven drying. The animals were provided with ad libitum water during the study, and water consumption in the CCR was recorded at the time of each observation using endurance multi-jet turbine water metres (RMC Zenner, Eagle Farm, QLD, Australia). The climate-controlled facility was equipped with cameras (K-guard CW214H; New Taipei City, Taiwan), with two cameras over each pen attached to a digital video recorder (LG, XQ-L900H; Yeouido-dong, Seoul, Republic of Korea) for surveillance of the animals.

### 2.3. Behaviour and Other Key Measurements

Respiratory behaviour was recorded 7 times a day, every 2 h from 0600 to 1800 h during TN and Recovery and every hour over 24 h during the HOT period. A team of five trained observers recorded respiration rate and panting score (PS). The respiration rate (RR) was determined by timing ten breaths from flank movements, converted into breaths per minute. The panting scores (PSs) of animals were visually scored based on a modified scale from 0 to 4.5, where PS 0 indicates no heat stress and PS 4.5 represents a severely heat-stressed animal [23]. From the video recordings during the CCR phase, the chewing rate while eating was determined by counting chews during one minute at the time of morning feed. These recordings were obtained on d 6 (TN), d 9–15 (HOT), and d 17–19 (Recovery).

From the video recordings, standing, lying, stepping of each limb, eating, ruminating, grooming, and scratching were recorded using event-logging [24] software (BORIS, version 6.0.4) for 24 h (5 min/h) on d 6 (TN); d 9, 11, 13, and 15 (HOT); and d 17–19 (Recovery) (Table 3). Head, ear, and tail positions were also recorded at 5 min intervals every h for 24 h. Not all behaviours could be observed at all times: accurate recording of ear position was not possible when the animal was ruminating, eating, drinking, scratching, or grooming; recording of head position was not possible during eating, drinking, grooming, or scratching, nor tail position during defecation, urination, eating, drinking, grooming, or scratching. At the end of the trial, after the animals had returned to the experimental feedlot from the climate control facility, the behaviour of the cattle was observed at 5 min intervals from 0900 h to 1700 h daily for their first two days in the feedlot.

### 2.4. Infrared Eye Temperature

Images of both eyes (IRT-Eye) of each animal were taken using an infrared thermal imager (FLK-Ti25 9 Hz, Fluke Corporation, Everett, WA, USA) at 0600, 1200, and 1800 daily for 6 (TN); d 9–15 (HOT); and d 17–19 (Recovery). The images of the animals were taken whilst located in the pen inside the climate control rooms at approximately 1m distance from the eye.

In order to maximise usefulness of thermal images, the IRT-Eye images were taken by capturing the object at a perpendicular angle to the infrared thermal camera. Only usable infrared thermal images (which comprised 84% of all images) were selected for analysis using an emissivity value of 0.98, while non-usable images (16%) were excluded. Minimum, maximum, and average eye temperatures from both right and left eyes were determined using a zone analysis marker; however, only the maximum eye temperatures within the zone analysis were used for further analysis, as this represents the best image from which to determine stress in cattle [25]. Mean eye temperature was calculated from infrared thermal eye temperature of both eyes ((right eye + left eye)/2). Infrared thermography images obtained were analysed using proprietary software (Fluke Smart View Software, version 4.3, Fluke Corporation, Everett, WA, USA).

### 2.5. Climatic Data

Climatic conditions (T_A_ and RH) inside each CCR were monitored at 10 min intervals using two temperature and humidity data loggers per room (HOBO UX100-011, Onset, MA, USA). A temperature humidity index (THI) was calculated using the following equation, adapted from Thom [26]:

THI = (0.8 × T_A_) + {[(RH/100) × (T_A_ − 14.4)} + 46.4] (1)

where RH = relative humidity in %, and T_A_ = ambient temperature in °C.

### 2.6. Statistical Analyses

Two animals (one at the start and a second towards the end of HOT) were removed from the experiment during the HOT period due to their inability to cope with hot conditions. However, the data obtained from these two animals was used for their duration in the experiment until they were removed.

The data obtained was analysed using the statistical software Minitab 18 (Minitab^®^, version 18.1 Inc., Chicago, IL, USA) for Windows. Two separate models were used to describe the data. First, a comparison between the TN and HOT periods, including animal ID as a random factor and the following fixed factors: dietary cohorts (D; finisher and substituted diets) and the treatment period [P; HOT and TN], as well as the interaction treatment period with diet.

Second, linear relationships between climatic conditions in the HOT period and the biological responses of feedlot cattle were determined using a mixed effects model, with animal identification (ID) as a random factor and the following fixed factors: cohorts (D, finisher, and substituted diets), treatment period [P, HOT, and Recovery], and day (d) of the experiment, nested within the treatment, as well as the interactions, diet × treatment period and diet x day. Data from the 1 d of recording in the TN was used as a covariate (Cov). The equation for the analysis was:

*Y_B_ = µ + D + P + d (P) + ID + (D* × *P) + (D* × *d (P)) + Cov + e* (2)

where $Y_{B}$ is the expected value for biological response variables, *μ* is the expected mean value for response variables when input variables = zero, the factors are as described above, and e is the random error associated with experimental observations.

Pairwise comparisons between treatment means were performed using Fisher’s test. Logarithm transformations (log_10_ + 1) were made for variables in order to achieve an approximate normal distribution of the residuals. When the proportion of zeros was more than 50% and a linear model produced residuals that were not normally distributed, the data was dichotomised into binary format according to whether they did or did not perform it each day and analysed by binary logistic regression using a logit model. Raised head position, raised ear position, and raised tail were analysed in this way. The behavioural events, downward ears and tucked tail were analysed using a chi-square goodness-of-fit test to estimate daily behavioural counts for each animal/day during each experimental period.

To investigate the association of behavioural responses with panting and respiration rates, Spearman’s rank correlation coefficients (r_s_) with a two-tailed level of significance (*p* < 0.05) were determined. This method was also used to investigate the relationship between their behaviour in the feedlot and the differential in the behaviour of cattle during the HOT period and that in the first thermoneutral period, i.e., a measure of the cattle that reacted most to the high temperatures. The Benjamini–Hochberg procedure was used to decrease false discovery rates, with a critical value for a false discovery rate of 0.25 [27,28].

## 3. Results

Two animals (one at the start and a second towards the end of the hot period) were removed from the experiment during the hot period due to their inability to cope with hot conditions. However, the data obtained from these two animals was used for their duration in the experiment until they were removed.

### 3.1. Stepping, Standing and Lying

The behaviour of the animals (*n* = 12) exposed to high temperatures (HOT), compared with the TN, are presented in Table 4. The stepping rates of all four limbs were greater in the HOT period than the TN period. Total stepping was approximately doubled. There was relatively more left- than right-limb stepping and back- than front-limb stepping in the HOT period than in the TN period. Standing time was greater, and lying was decreased in HOT compared with TN.

The behaviour of the animals (*n* = 12) exposed to the HOT and Recovery periods are compared in Table 5. The stepping rates of all four limbs stepping were greater in HOT than Recovery. No significant difference was observed for back limbs relative to front limbs and left relative to right limbs in stepping in the HOT period compared with the Recovery period. Standing time was greater, and lying was decreased in HOT compared with the Recovery period.

### 3.2. Ears

The ears were more in backward and forward orientations in the HOT than TN periods (Table 4), but there was no difference in the axial position of the ears. The ears were backward much more in HOT than the Recovery period (Table 5). Ears forward and in the axial position were not significantly different in the HOT and Recovery periods. In the binary logistic regression of ears raised, approximately the same proportion of cattle were observed to hold their ears raised at least once a day in the TN period and in the HOT treatment (42 and 41%, respectively), which then declined significantly in the Recovery period (10%) (OR 0.12; CI 0.03–0.48; *p* = 0.003). No significant differences in downward ears were observed during these three different periods (chi-square, 2.58; *p* = 0.28).

### 3.3. Head

Head down and neutral positions were not different in the HOT period compared with the TN (Table 4). Head down was more commonly observed for cattle in HOT than in the Recovery period (Table 5). No differences in the numbers of cattle with a raised head position was observed in the different periods (chi-square, 5.41; *p* = 0.07).

### 3.4. Tail

Cattle held their tails more in a vertical position in the HOT period than in TN, but no difference in tail swishing was observed (Table 4). Cattle held their tails more in a vertical position and with less swishing in the HOT period than in the Recovery period (Table 5). No differences in the raised (chi-square, 2.80; *p* = 0.3) and tucked tail position (chi-square, 2.58; *p* = 0.3) were observed during different treatment periods.

### 3.5. Nutritional Behaviours

Rumination time was greatly reduced and chewing rate slightly reduced during the HOT period compared with TN. Eating time and DM intakes were approximately halved for cattle in the HOT period compared with TN. The chewing rate was reduced by approximately 15% for cattle during the HOT period compared with the Recovery period. The rumination time, eating time, and DM intake were approximately halved for steers in the HOT period compared with the Recovery period.

### 3.6. Scratching and Grooming

Scratching was much reduced in the HOT period compared with TN (Table 4), although grooming was not different. Compared with the Recovery period, grooming and scratching were much reduced in the HOT period (Table 5).

### 3.7. Respiration and Behaviour Correlations with Panting, Respiration, and Eye Temperature

Respiration rate and panting scores approximately doubled during the HOT period, compared with TN (Table 4). Respiration rate and panting scores were approximately doubled during the HOT period compared with the Recovery period (Table 5). Spearman’s rank correlation of differences in the HOT—TN means of behaviours with the differences in the HOT—TN means of physiological parameters showed that respiration rates (correlation coefficient (CC −0.66; *p* = 0.03) and panting scores (CC −0.70; *p* = 0.02) were both negatively associated with forward ear position.

### 3.8. Infrared Eye Temperature and Correlation with Behaviour

The infrared eye temperatures of cattle (*n* = 12) exposed first to TN and then to the high temperature environment are compared in Table 4. The infrared thermographic temperature (°C) of right, left, and mean infrared eye temperatures of both eyes in the cattle was greater in the HOT period than TN. No difference was observed in the infrared R/L eye ratio (°C) of the cattle between the HOT and TN periods. The infrared eye temperature °C (right eye, left eye, and mean infrared eye) of the cattle was greater in HOT than in the Recovery period (Table 5). The infrared R/L eye ratio was not affected by the periods.

Spearman’s rank correlation of differences in the HOT—TN means of behaviours with the differences in the HOT—TN means of physiological parameters showed that mean infrared eye temperatures were negatively correlated with front left-limb stepping (CC −0.72; *p* = 0.01) and forward ear position (CC −0.60; *p* = 0.05).

### 3.9. Feedlot Behaviour and Correlations with Behaviour in the HOT Period

At the end of the experiment, the animals were moved to the feedlot from CCR and their behavioural response in the feedlot was observed as the recovery phase, as reported in the methodology section. Mean values (±SEM) as a proportion of time for the behaviours recorded in the feedlot were standing (0.69 ± 0.022), lying (0.29 ± 0.016), eating (0.013 ± 0.0042), ruminating (0.054 ± 0.005), grooming (0.029 ± 0.0055), scratching (0.0069 ± 0.0027), raised head (0.059 ± 0.011), neutral head (0.78 ± 0.014), downward head (0.084 ± 0.0099), raised ear (0.075 ± 0.01), downward ears (0.17 ± 0.0066), axial ear (0.17 ± 0.008), forward ear (0.11 ± 0.01), backward ear (0.35 ± 0.0088), vertical tail (0.5 ± 0.01), raised tail (0.05 ± 0.0085), tucked tail (0.0025 ± 0.0014), and swishing tail (0.37 ± 0.0072).

The association of these behaviours in the feedlot with the differentials in behaviour means (HOT—TN) of cattle from the first thermoneutral period to the HOT period (i.e., those that responded behaviourally most to the high temperatures) is presented in Table 6. Animals who increased time standing during the HOT period spent the least time with their ear in an axial position in the feedlot. Conversely, those spending more time lying in the HOT period spent more time with their ears in axial position in the feedlot. Those increasing backward ear positions the most in the HOT period spent the least time with their head in a neutral position in the feedlot. The animals with increased time spent with forward ears in the HOT spent the least time with their ears in backward direction in the feedlot. Those reducing their eating time most in the HOT period spent the least time with their head downwards and the most time with a vertical tail, scratching, and with forward and downward ears in the feedlot.

## 4. Discussion

High environmental temperatures initiate behavioural responses to maintain homeostasis (thermal balance) in the feedlot cattle. Among the various responses, increased panting, respiration, standing, and shade-seeking behaviours are well reported in cattle experiencing heat stress [11,18].

### 4.1. Standing, Lying, and Stepping

An increase in standing time during hot environmental conditions and decreased lying time have been associated with discomfort in cattle [29,30]. Shultz [31] also reported this while investigating the impact of shade on standing behaviour for cattle experiencing high environmental temperatures. The importance of increased standing time for heat dissipation from the body surface through evaporation and convective heat exchange is well established [29,30]. Thus, increased standing time in the current study further confirms standing as an adaptive behaviour to heat stress.

The hot environmental conditions also caused increased stepping of all four limbs in the HOT period (Table 4 and Table 6). This increased stepping during the hot environment may reflect irritation and discomfort, and it has been reported in previous reports of increased stepping in cattle and sheep during other stressful conditions [14,32] (human–animal interactions and tick lesions, Rousing et al. [14]; novel stimuli, Amira et al. [15], pain and hoof lesions [33,34]). However, when observed in sheep in response to floor movement, Navarro et al. [35] suggested that increased stepping reflected balance correction. However, increased stepping during the period of high environmental temperatures may represent cattle attempt to escape [14,15] from stressful environment.

Cattle demonstrate preferences for the side of the body employed to respond to novel stimuli [15]. In animals, the expression of right- and left-handedness (from a limb perspective) is contralateral to the left and right brain hemispheres, which process information as proactive and reactive behavioural responses [36,37], respectively. In the current study, this lateralised stepping behaviour in beef cattle during HOT conditions represents a key novel behaviour considered to be a response to thermal stress (Table 4). Cattle may be doing this behaviour in response to different sorts of stressors, not exclusively to thermal stress. Animals expressing left-handedness (from a limb perspective) are likely to be more stressed [36,38], as this response is being processed through the right brain hemisphere, which processes fight-or-flight reactions.

Cattle also stepped more with the back limbs relative to the front limbs in HOT than in the TN period (Table 4). This could be related to differential weight distribution between front and back limbs, with more weight on the front legs due to head weight [34]. The front limb also remains less mobile, as cattle use it more to steer the load and for body support [39,40]. The greater back-limbs stepping during the HOT period probably reflects the fact that cattle require a strong thrust from the back legs to initiate any escape attempts [39] during a period of high environmental temperatures.

### 4.2. Ears

Different ear positions (backward, forward, downwards, raised, and axial ears) were observed during heat stress as a potential non-invasive measure to be incorporated into farm animal welfare assessments. These ear positions are indicative of positive and negative emotions in animals [41].

Consistently, a more backward ear position was observed in cattle during hot conditions, suggesting a negative emotion for cattle in the HOT period, as proposed by Boissy et al. [42]. When observed in the feedlot, these animals with increased backward ear position in HOT spent the least time with their head in a neutral position in the feedlot, indicating a persistent effect of heat stress in these animals. The neutral head position is well reported as an indication of a relaxed animal [15].

A backward ear position indicates fear, discomfort, pain, and stressful situations [42,43], while forward, downward, and axial ear positions have been associated with relaxed cattle [16,43]. It was associated with pain responses in cattle by Gleerup et al. [43], and in sheep, it has been observed during uncontrollable and unpleasant situations that elicit a desire to escape [42]. The backward ear position is also the predominant orientation during feeding and grooming (brushing) activities [16], possibly to protect the inner ear; however, in the current study, ear positions during eating, grooming, and scratching were not recorded.

Although a relatively small increase in the time spent with forward ear position was observed in HOT compared with TN, that difference is not reflected in any decline in Recovery after the HOT period. Contrary to earlier findings [4], this small increase in the time spent with forward ear positioning in the current study could be attributed to a comparatively lower temperature in the current study during the HOT period. Those animals that spent the most time with forward ear positions in HOT also spent the least time with backward ear positions in the feedlot. Further, a negative association of forward ear position with respiration, panting, and infrared eye temperature confirms that the most heat-stressed animals spent the least time with forward ear positions. This is probably because these behaviours are associated with backward, rather than forward, ear positions.

A raised ear position indicates fear, fight, and flight during stressful situations when exposed to novel stimuli [42]. Raised ear position was not different in HOT and TN; however, there was a significant decline in the Recovery period. The decline in raised ear position could be attributed to each ear moving more frequently and independently in the Recovery period. Axial ears, with each ear moving frequently, are an indication of a relaxed animal [43]. Animals that spent more time standing during HOT spent the least time with axial ears in the feedlot, and this was increased in the feedlot for those who spent most time lying. The decline in axial ears in the feedlot is an indication of persistence of stress induced by the hot conditions in CCR.

### 4.3. Head

The head has an important role for maintaining body balance in quadrupeds [39,44], but its position has also been associated with emotions and stress-related responses [15,43,45]. In the current study, the time spent with a downward head position was increased in the HOT period compared with Recovery. The downward head position can be attributed to an effort to dissipate heat [4] through their trachea better, which may require cattle to extend their neck and lower their head below their withers. Alternatively, it could be an attempt to inhale cooler air, suggesting that the behaviour is adaptive. This could be different in highly stocked pens, where hot air is trapped at animal level.

### 4.4. Tail

Different tail positions have been studied with reference to various stimuli in cattle [15,16]. In the current study, we observed that cattle spent longer with their tail held in a vertical tail position and engaged in less swishing of the tail. Vertical and swishing are the most observed tail positions in cattle, indicating relaxed behaviour [16]. However, the decline in swishing tail during hot conditions may function to avoid energy utilisation and heat generation [46].

### 4.5. Nutritional Behaviours

A major decline in the proportion of time spent eating, chewing while eating, ruminating, and dry matter intake were recorded during the HOT period, with the cattle recovering afterwards. The initiation of heat dissipating mechanisms during hot conditions therefore switches energy from routine behavioural activities such as eating, ruminating, and self-grooming [46] to other activities like increased sweating, panting, and respiration rate [2]. Feed intake is responsible for approximately 3 to 8% of heat production in cattle [47]. Animals that spent the least time eating during the HOT period spent the most time scratching, with their tail vertical, ears down or forward, and the least time with their head in a downward position in the feedlot, indicating that they had at least partially recovered. These change in nutritional behaviours confirm the adaptive nutritional responses of the animals to achieve homeostasis.

### 4.6. Self-Grooming

Self-grooming declines during heat load conditions, perhaps due to declining body energy resources in dairy cows [48] and calves [49]. Consistently, a reduction in self-grooming was observed in the current experiment during the HOT period, compared with later, supporting the inclusion of this behaviour in the suite of cattle responses to heat.

### 4.7. Respiration

Alterations in respiratory dynamics (RR and PS) serve as the most common early signs of increasing heat stress, as cattle attempt to maintain homeostasis by dissipating excessive heat load during high environmental temperatures [3]. Respiration and panting increased during the HOT period compared with the Recovery and TN periods [12,18]. This increasing respiration rate reflects the imbalance between heat accumulated and dissipated in heat-stressed animals [23]. Increased respiration helps to dissipate approximately 30% of total heat dissipated, being primarily influenced by changes in ambient temperature, after a short lag time (1–2 h), making it a prime candidate for an easily measurable indicator of heat stress [12,18].

### 4.8. Infrared Eye Temperature

The rise in core body temperature during heat stress provokes the animal to increase its peripheral circulation [18] to dissipate accumulated heat from the body. This change in the blood circulation leads to increased heat dissipation from the body surfaces including the eyes, especially around the *caruncula lacrimalis,* which is innervated by rich capillary beds [19]. Infrared eye temperature, captured as eye thermograms, can be a promising and reliable method for assessing cattle responses and well-being during hot days, with the additional advantage of minimum stress and quick measurement [2].

Increases in the infrared thermographic temperature (°C) of the right, left, and mean eye temperatures were observed in HOT as compared with the TN and Recovery periods (Table 4 and Table 6). A negative association of IRT eye temperature also existed with front left-limb stepping and forward ear position. The decline in stepping with increasing IRT eye temperature could be the result of a decline in front-limb stepping, as back-limb stepping was increased during the HOT period. This may be attributed to a lack of energy or the animals’ attempting to lower heat generation during the HOT period [32,46]. However, decreased time spent with forward ears indicates stress from the heat load, as discussed above in relation to ear positions. No differences in R/L eye ratios were observed during hot conditions. These consistent findings regarding changes in infrared eye temperature supports the importance of IRT eye measurements as a non-invasive tool to assess cattle responses to hot environmental conditions.

### 4.9. Limitations

The environmental conditions in the climate-controlled rooms were well controlled, and each animal was individually kept in a pen; however, in the feedlot, the environmental conditions were not controlled, which limited the recording of detailed behaviour. Furthermore, there is a possibility that some behaviours may have been affected because of these differences in animal management in the feedlot and climate-controlled rooms. However, the behavioural pattern of the heat-stressed animals in the feedlot is presented and discussed to enhance our understanding of recovery after a hot environmental period.

The limited sample size (*n* = 12) should also be considered as one of the limitations of the study; therefore, the current results should be considered as the preliminary validation of the behavioural and physiological responses to high environmental temperatures. Carefully considering ethical issues, future studies with more animals of different breeds, species, and physiological stages can further validate the current findings.

## 5. Conclusions

The study provided validation evidence that cattle exposed to high environmental temperatures spendmore time standing, exhibited increased stepping. There was a lateralised stepping responses, which appeared in cattle to reflect discomfort, depression, and/or frustration, while the back limb response, suggests an escape response during hot environmental conditions. The hot environmental conditions also reduced the normal maintenance behaviours including eating, chewing, ruminating, grooming, and scratching, as observed in one of our previous experiments.

Changes in infrared eye temperature were consistently observed, indicating the importance of IRT eye measurements to assess cattle responses to hot environmental conditions. It is concluded that cattle express consistent changes in behaviour and physiology in response to high environmental temperatures that could be used to detect the animals most affected, and that some, but not all, responses appearing to be adaptive.

## Figures and Tables

**Table 1:** The ambient temperature, relative humidity, and temperature humidity index for cattle (*n* = 12) in the climate control facility.

Day	Treatment Phase	Min T_A_ (°C)	Max T_A_ (°C)	Mean T_A_ (°C)	Min RH (%)	Max RH (%)	Mean RH (%)	Min THI	Max THI	Mean THI
1	TN (ACC)	18.9	21.2	19.9	57.6	93.8	68.9	64.8	69.7	66.1
2	TN (ACC)	19.0	21.2	20.0	39.5	91.6	68.4	63.4	66.2	66.2
3	TN (ACC)	19.1	22.5	20.2	52.6	91.9	69.4	65.1	71.4	66.5
4	TN	19.3	21.3	20.3	54.4	89.0	68.6	65.4	68.9	66.6
5	TN	19.6	21.3	20.4	53.9	89.6	68.1	65.6	69.6	66.8
6	TN	19.7	21.4	20.4	53.2	90.2	66.9	65.3	69.8	66.8
7	TN	19.1	21.5	20.3	55.1	91.2	68.6	64.9	70.0	66.7
8	TP1	19.2	25.1	20.9	52.6	88.2	67.8	64.8	74.3	67.5
Transition to HOT phase occurs from 2100 h on d 8										
9	HOT	24.7	36.7	31.2	48.9	90.0	73.8	73.1	90.4	83.5
10	HOT	27.8	36.8	31.6	46.9	87.0	73.7	78.9	90.4	84.1
11	HOT	27.0	36.9	31.6	47.2	92.4	73.2	77.9	90.8	84.1
12	HOT	28.1	37.3	31.8	48.7	87.1	72.4	78.9	89.9	84.1
13	HOT	28.1	37.2	31.8	47.4	85.7	72.5	78.6	90.6	84.2
14	HOT	25.7	35.1	30.4	46.9	90.4	72.3	74.9	87.5	82.1
15	HOT	27.1	35.3	30.4	47.4	87.1	72.5	77.4	88.8	82.1
16	TP2	19.4	28.7	21.2	49.9	87.2	64.8	65.1	80.6	67.8
17	Recovery	19.3	21.1	20.0	53.0	89.1	65.6	65.2	68.8	66.1
18	Recovery	19.3	21.1	20.1	56.9	88.3	65.6	64.9	68.4	66.2
19	Recovery	19.2	21.0	20.1	59.8	88.2	67.7	65.0	68.4	66.2
20	Recovery	19.3	20.9	19.9	61.6	97.3	68.7	65.0	68.9	66.1
21	Recovery	19.1	21.5	19.9	61.8	90.3	74.0	64.9	69.7	66.4
22	Recovery	18.7	21.6	21.0	25.0	88.6	70.0	62.8	69.6	67.7

T_A_: ambient temperature (°C); RH: relative humidity; THI: temperature humidity index; ACC: acclimatisation to climate-controlled facility; TN: thermoneutral conditions before high temperature treatment; TP1 and 2: transition phases to and from hot conditions; HOT: high temperature treatment; Recovery: thermoneutral conditions after high temperature treatment as a recovery period.

**Table 2:** Diet ingredients and nutrient composition of diets fed to cattle at the experimental facility.

Item	Finisher Diet	Substituted Diet
Ingredients, % of diet		
Sub-batch grain mix *	86.8	78.7
Whole cottonseed	9.0	9.0
Alfalfa (Lucerne) hay	4.2	12.3
Nutrient composition		
DM, g/kg fresh weight	887	886
ADF, g/kg DM	119	177
NDF, g/kg DM	229	253
NE_g_, MJ/kg DM	30	30
ME, MJ/kg DM	132	131
DE, MJ/kg DM	163	162
Crude Fibre, g/kg DM	87	124
Nitrogen Free Extract, g/kg DM	678	685
Fat, g/kg DM	46	43
Feed Digestibility, g/kg DM	861	868
Digestible DM, g/kg DM	763	769
Digestible Protein g/kg DM	130	131
Starch, g/kg DM	432	432

* Sub-batch grain mix: Feedlot pellet ^#^ 9.2%, steam-rolled barley 89.2%, vegetable oil 1.6%. ^#^ The Feedlot pellet contains: Millrun wheat 55.9%, ammonium sulphate 2.6%, dry-rolled wheat 12.5%, calcium carbonate (limestone) 15.6%, Rumensin 100—0.3%, magnesium oxide 0.7%, Availa zinc 100—0.34%, vegetable oil 3.1%, salt, plain (NaCl) 2.8%, urea 5.7%, vitamin A 500—0.009%, vitamin E 0.057%, XFE-Select L 0.385%.

**Table 3:** Ethogram for recorded behaviours for cattle (*n* = 12) housed in individual pens in the climate-controlled facility.

Item	Description
Respiration rate	Time taken for 10 breaths, determined from flank movement
Panting score	Animal visually scored for extent of panting based on 0 to 4.5 score scale [23]
Standing	Animal standing with limb positioned upright
Lying	Animal resting on the floor with its limbs laterally or sternally recumbent
Eating	Animal consuming feed at the trough
Chews while eating	Chews counted during one minute at the time of morning feed
Rumination	Animal chewing a bolus or regurgitating bolus
Self-grooming	Grooming or scratching, behaviour performed by animal on itself
Grooming	Animal licking any part of the body or striking one part with another part of the body
Scratching	Animal rubbing or striking any part of the body against fixture of the pen
Ear positions	
Ear raised	Both ears being held upright above the neck with the ear pinnae facing forwards or to the side
Ear forward	Both ear pinnae being directed forwards in front of the focal animal and the ear being held horizontally
Ear backward	Both ears being held backwards on the animal’s head
Ear downward	Both ears being loosely hung downwards, falling perpendicular to the head
Ear specific	Ear pinnae (right and left) oriented in opposite directions, or perpendicular to head rump axis, failing to satisfy raised, forward, backward, and downward ear positions
Ear axial	Ears straight out to the side, perpendicular to the head-rump axis [16]
Head positions	
Head raised	The head held upright above withers or body top-line
Head neutral	The head held horizontally at the level of withers or body top-line
Head downwards	The head held downwards below withers or body top-line
Stepping	
Front right (FR) limb	Animal raising a front right limb and replacing it forthwith on the surface of pen
Front left (FL) limb	Animal raising a front left limb and replacing it forthwith on the surface of pen
Back right (BR) limb	Animal raising a back right limb and replacing it forthwith on the surface of pen
Back left (BL) limb	Animal raising a back left limb and replacing it forthwith on the surface of pen
Tail positions	
Tail raised	Tail held in a fixed position, at 45 degrees from the vertical
Tail vertical	Tail hanging downward in vertical line from the body with no movements
Tail swishing	Swift movement of the tail in any direction around the hind quarters from its base in a side-to-side flicking manner
Tail tucked	Tail held tightly pressed in a fixed position against the rump, with a tip of the tail tucked in the hind limb

Adapted from Idris et al. [2,4].

**Table 4:** Behavioural and physiological responses of cattle (*n* = 12) exposed to high temperatures (HOT treatment) or an initial thermoneutral period before high temperature treatment (TN).

Behaviour	Periods		SED	F-Value (1, 43 d.f. ^†^)	*p*-Value
	TN	HOT			Period (P)
Stepping					
FR limb, Log_10_ + 1 counts/5 min (counts/5 min)	0.66 ^b^ (3.55)	0.85 ^a^ (6.08)	0.101	11	0.002
FL limb, Log_10_ + 1 counts/5 min (counts/5 min)	0.58 ^b^ (2.80)	0.79 ^a^ (5.17)	0.0991	14	≤0.001
BR limb, Log_10_ + 1 counts/5 min (counts/5 min)	0.64 ^b^ (3.38)	0.89 ^a^ (6.69)	0.0991	19	≤0.001
BL limb, Log_10_ + 1 counts/5 min (counts/5 min)	0.59 ^b^ (2.86)	0.84 ^a^ (5.92)	0.101	20	≤0.001
Total Stepping, Log_10_ + 1 counts/5 min (counts/5 min)	1.12 ^b^ (12.28)	1.39 ^a^ (23.66)	0.113	18	≤0.001
R/L limb ratio, Log_10_ + 1 (ratio of counts/5 min)	0.35 ^a^ (1.24)	0.33 ^b^ (1.15)	0.0159	4	0.05
F/B limb ratio, Log_10_ + 1 (ratio of counts/5 min)	0.31 ^a^ (1.05)	0.28 ^b^ (0.91)	0.0196	8	0.01
Standing/Lying					
Standing, Log_10_ + 1 prop. time (prop. time)	0.12 ^b^ (0.32)	0.16 ^a^ (0.46)	0.0237	11	0.002
Lying, Log_10_ + 1 prop. time (prop. time)	0.23 ^a^ (0.68)	0.18 ^b^ (0.53)	0.0248	8.98	0.004
Ears, head, and tail					
Ear backward, Log_10_ + 1 prop. time (prop. time)	0.14 ^b^ (0.37)	0.17 ^a^ (0.47)	0.0157	11	0.002
Ear forward, Log_10_ + 1 prop. time (prop. time)	0.032 ^b^ (0.077)	0.046 ^a^ (0.11)	0.0103	5	0.03
Ear axial, Log_10_ + 1 prop. time (prop. time)	0.108 (0.28)	0.109 (0.29)	0.0259	0.01	0.9
Head downward, Log_10_ + 1 prop. time (prop. time)	0.018 (0.043)	0.024 (0.057)	0.0113	3	0.11
Head neutral, Log_10_ + 1 prop. time (prop. time)	0.28 (0.89)	0.28 (0.89)	0.0121	0.33	0.9
Tail vertical, Log_10_ + 1 prop. time (prop. time)	0.27 ^b^ (0.87)	0.29 ^a^ (0.93)	0.00889	6.56 (1, 50)	0.01
Tail swishing, Log_10_ + 1 prop. time (prop. time)	0.0067 (0.016)	0.0059 (0.014)	0.00637	0.14 (1, 41)	0.7
Oral behaviours					
Groom, Log_10_ + 1 prop. time (prop. time)	0.0052 (0.012)	0.0039 (0.0091)	0.00254	0.80	0.4
Scratch, Log_10_ + 1 prop. time (prop. time)	0.0028 ^a^ (0.0065)	0.0012 ^b^ (0.0028)	0.00138	4	0.05
Rumination, Log_10_ + 1 prop. time (prop. time)	0.075 ^a^ (0.19)	0.027 ^b^ (0.064)	0.0109	62 (1, 42)	≤0.001
Eating, Log_10_ + 1 prop. time (prop. time)	0.026 ^a^ (0.061)	0.0036 ^b^ (0.0083)	0.00595	42	≤0.001
Chewing while eating, Log10 + 1 chews/minute (chews/minute)	1.98 ^a^ (94.94)	1.89 ^b^ (77.16)	0.0178	78 (1, 59)	≤0.001
Dry matter intake, Log_10_ + 1 kg/day (kg/day)	1.07 ^a^ (10.83)	0.82 ^b^ (5.56)	0.0781	30 (1, 68)	≤0.001
Respiration rate, Log_10_ + 1 breaths/min (breaths/min)	1.84 ^b^ (68.66)	2.08 ^a^ (119.23)	0.0214	395 (1, 75)	≤0.001
Panting Score (PS), Log_10_ + 1 PS score (PS score)	0.31 ^b^ (1.02)	0.46 ^a^ (1.90)	0.0133	450 (1, 75)	≤0.001
Infrared eye temperature (°C)					
Right eye temperature, Log_10_ + 1 °C (°C)	1.57 ^b^ (35.73)	1.59 ^a^ (37.91)	0.00982	46 (1, 217)	≤0.001
Left eye temperature, Log_10_ + 1 °C (°C)	1.57 ^b^ (35.73)	1.59 ^a^ (37.99)	0.00982	55 (1, 213)	≤0.001
Mean eye temperature, Log_10_ + 1 °C (°C)	1.57 ^b^ (35.81)	1.59 ^a^ (37.91)	0.00981	64 (1, 239)	≤0.001
Right/left eye ratio, Log_10_ + 1 (ratio of eye temperature; °C)	0.30 (1.00)	0.30 (0.99)	0.00981	0.54 (1, 192)	0.50

FR: Front right-limb stepping; FL: front left-limb stepping; BL: back left-limb stepping; BR: back right-limb stepping; Log_10_ + 1: logbase10 + 1; R/L limb ratio: Right/Left limb stepping count ratio; F/B limb ratio: Front/Back limb stepping count ratio; SED: standard error of the difference between two means; HOT: high temperature treatment period, d 9–15; TN: thermoneutral period before high temperature treatment, d 6; ^†^ treatment: error degrees of freedom; P: period; Superscripts ^a,b^; means within each variable with different superscripts are different at *p* ≤ 0.05 by Fisher’s test.

**Table 5:** Behavioural and physiological responses of cattle (*n* = 12) exposed to high temperatures (HOT treatment) or a thermoneutral Recovery period.

Behaviour	Periods		SED	F-Value (1, 53 d.f. ^†^)	*p*-Value
	HOT	Recovery			Period (P)
Stepping					
FR limb, Log_10_ + 1 counts/5 min (counts/5 min)	0.85 ^a^ (6.05)	0.70 ^b^ (3.97)	0.0768	22	≤0.001
FL limb, Log_10_ + 1 counts/5 min (counts/5 min)	0.79 ^a^ (5.15)	0.66 ^b^ (3.53)	0.0745	18	≤0.001
BR limb, Log_10_ + 1 counts/5 min (counts/5 min)	0.89 ^a^ (6.73)	0.74 ^b^ (4.55)	0.0756	20	≤0.001
BL limb, Log_10_ + 1 counts/5 min (counts/5 min)	0.84 ^a^ (5.93)	0.701 ^b^ (4.02)	0.0783	18	≤0.001
Total Stepping, Log_10_ + 1 counts/5 min (counts/5 min)	1.39 ^a^ (25.55)	1.23 ^b^ (15.98)	0.0849	21	≤0.001
R/L limb ratio, Log_10_ + 1 (ratio of counts/5 min)	0.332 (1.15)	0.327 (1.12)	0.0107	1.25 (1, 51)	0.3
F/B limb ratio, Log_10_ + 1 (ratio of counts/5 min)	0.279 (0.90)	0.277 (0.89)	0.0191	0.03 (1, 51)	0.9
Standing/Lying					
Standing, Log_10_ + 1 prop. time (prop. time)	0.16 ^a^ (0.45)	0.14 ^b^ (0.38)	0.0202	6	0.02
Lying, Log_10_ + 1 prop. time (prop. time)	0.19 ^b^ (0.53)	0.21 ^a^ (0.61)	0.0208	5	0.02
Ears, head, and tail					
Ear backward, Log_10_ + 1 prop. time (prop. time)	0.17 ^a^ (0.47)	0.14 ^b^ (0.39)	0.0146	15	≤0.001
Ear forward, Log_10_ + 1 prop. time (prop. time)	0.046 (0.11)	0.038 (0.091)	0.00987	3.4	0.07
Ear axial, Log_10_ + 1 prop. time (prop. time)	0.11 (0.29)	0.12 (0.31)	0.0175	1.2	0.3
Head downward, Log_10_ + 1 prop. time (prop. time)	0.027 ^a^ (0.063)	0.016 ^b^ (0.038)	0.00982	6	0.02
Head neutral, Log_10_ + 1 prop. time (prop. time)	0.276 (0.89)	0.275 (0.88)	0.00690	0.25 (1, 49)	0.6
Tail vertical, Log_10_ + 1 prop. time (prop. time)	0.29 ^a^ (0.93)	0.28 ^b^ (0.88)	0.0056	17 (1, 51)	≤0.001
Tail swishing, Log_10_ + 1 prop. time (prop. time)	0.0064 ^b^ (0.015)	0.012 ^a^ (0.027)	0.00473	7 (1, 57)	0.01
Oral behaviours					
Groom, Log_10_ + 1 prop. time (prop. time)	0.0046 ^b^ (0.011)	0.016 ^a^ (0.037)	0.00352	57	≤0.001
Scratch, Log_10_ + 1 prop. time (prop. time)	0.0014 ^b^ (0.0031)	0.0046 ^a^ (0.011)	0.00229	11 (1, 54)	0.001
Rumination, Log_10_ + 1 prop. time (prop. time)	0.027 ^b^ (0.063)	0.044 ^a^ (0.11)	0.00999	17 (1, 52)	≤0.001
Eating, Log_10_ + 1 prop. time (prop. time)	0.0027 ^b^ (0.0063)	0.0058 ^a^ (0.013)	0.00329	5 (1, 51)	0.04
Chewing while eating, Log_10_ + 1 chews/minute (chews/minute)	1.89 ^b^ (76.63)	1.96 ^a^ (90.20)	0.0162	87 (1, 58)	≤0.001
Dry matter intake, Log_10_ + 1 kg/day (kg/day)	0.81 ^b^ (5.52)	1.004 ^a^ (9.09)	0.0514	91 (1, 72)	≤0.001
Respiration rate, Log_10_ + 1 breaths/min (breaths/min)	2.07 ^a^ (116.50)	1.65 ^b^ (43.67)	0.0191	3214 (1, 77)	≤0.001
Panting Score (PS), Log_10_ + 1 PS score (PS score)	0.46 ^a^ (1.87)	0.23 ^b^ (0.71)	0.0156	1393 (1, 77)	≤0.001
Infrared eye temperature (°C)					
Right eye temperature, Log_10_ + 1 °C (°C)	1.59 ^a^ (37.91)	1.57 ^b^ (35.81)	0.00866	86 (1, 155)	≤0.001
Left eye temperature, Log_10_ + 1 °C (°C)	1.59 ^a^ (38.17)	1.57 ^b^ (35.81)	0.00808	141 (1, 185)	≤0.001
Mean eye temperature, Log_10_ + 1 °C (°C)	1.59 ^a^ (38.08)	1.57 ^b^ (35.90)	0.00831	179 (1, 261)	≤0.001
Right/left eye ratio, Log_10_ + 1 °C (ratio of eye temperature; °C)	0.30 (0.99)	0.30 (1.00)	0.00101	0.06 (1, 100)	0.8

FR: Front right-limb stepping; FL: front left-limb stepping; BL: back left-limb stepping; BR: back right-limb stepping; Log_10_ + 1: logbase10 + 1; R/L limb ratio: Right/Left limb stepping count ratio; F/B limb ratio: Front/Back limb stepping count ratio; SED: standard error of the difference between two means; HOT: high temperature treatment period, d 9–15; Recovery: thermoneutral period after high temperature treatment, day 17–19; ^†^ treatment: error degrees of freedom; P: period; Superscripts ^a,b^; means within each variable with different superscripts are different at *p* ≤ 0.05 by Fisher’s test.

**Table 6:** Spearman’s correlations (*p* ≤ 0.05) between behaviour response to thermal stress (HOT period minus TN period) with their behaviour in the feedlot.

Behaviour in the Hot Period—TN Period	Differential Mean ± SE (Prop. Time) for the 2 Periods	Feedlot Behaviour	Correlation Coefficient Between the Differential and the Feedlot Behaviour	*p*-Value
Standing	0.14 ± 0.03	Ear axial	−0.859	≤0.001
Lying	0.14 ± 0.03	Ear axial	0.841	0.001
Ear backwards	0.101 ± 0.04	Head neutral	−0.673	0.02
Ear forward	0.031 ± 0.01	Ear backwards	−0.663	0.02
Eat	−0.057 ± 0.02	Ear downwards	0.71	0.01
Eat	−0.057 ± 0.02	Head downwards	−0.639	0.034
Eat	−0.057 ± 0.02	Ear forward	0.636	0.035
Eat	−0.057 ± 0.02	Scratch	0.604	0.049
Eat	−0.057 ± 0.02	Tail vertical	0.603	0.05

## Data Availability

The original contributions presented in this study are included in the article. Further inquiries can be directed to the corresponding author.

## References

[1] Brown-Brandl T.M. (2018). Understanding heat stress in beef cattle. Rev. Bras. Zootec..

[2] Idris M., Sullivan M., Gaughan J.B., Phillips C.J.C. (2024). The Relationship between the Infrared Eye Temperature of Beef Cattle and Associated Biological Responses at High Environmental Temperatures. Animals.

[3] Nienaber J.A., Hahn G.L. (2007). Livestock production system management responses to thermal challenges. Int. J. Biometeorol..

[4] Idris M.M., Gaughan J.B., Phillips C.J.C. (2024). Behavioural responses of beef cattle to hot conditions. Animals.

[5] Senft R., Rittenhouse L. (1985). A model of thermal acclimation in cattle. J. Anim. Sci..

[6] Gaughan J., Mader T., Holt S., Sullivan M., Hahn G. (2010). Assessing the heat tolerance of 17 beef cattle genotypes. Int. J. Biometeorol..

[7] Scharf B.A. (2008). Comparison of Thermoregulatory Mechanisms in Heat Sensitive and Tolerant Breeds of *Bos taurus* Cattle. Master’s Thesis.

[8] Tucker C.B., Coetzee J.F., Stookey J.M., Thomson D.U., Grandin T., Schwartzkopf-Genswein K.S. (2015). Beef cattle welfare in the USA: Identification of priorities for future research. Anim. Health Res. Rev..

[9] Blackshaw J.K., Blackshaw A. (1994). Heat stress in cattle and the effect of shade on production and behaviour: A review. Anim. Prod. Sci..

[10] Hahn G., Parkhurst A., Gaughan J. (1997). Cattle respiration rate as a function of ambient temperature. Am. Soc. Agric. Eng..

[11] Al-Hosni Y. (2019). Physiological and Rumen Microbial Responses of Angus Cattle (*Bos taurus*) Following Exposure to Heat Load. Ph.D. Thesis.

[12] Brown-Brandl T., Eigenberg R., Nienaber J., Hahn G.L. (2005). Dynamic response indicators of heat stress in shaded and non-shaded feedlot cattle, Part 1: Analyses of indicators. Biosyst. Eng..

[13] Ratnakaran A.P., Sejian V., Sanjo Jose V., Vaswani S., Bagath M. (2017). Behavioral responses to livestock adaptation to heat stress challenges. Asian J. Anim. Sci..

[14] Rousing T., Bonde M., Badsberg J.H., Sørensen J.T. (2004). Stepping and kicking behaviour during milking in relation to response in human–animal interaction test and clinical health in loose housed dairy cows. Livest. Prod. Sci..

[15] Amira A.G., Gareth P.P., Jashim U., Eloise R., Harriet D., Clive J.P. (2018). A forced lateralisation test for dairy cows and its relation to their behaviour. Appl. Anim. Behav. Sci..

[16] deOliveira D., Keeling L.J. (2018). Routine activities and emotion in the life of dairy cows: Integrating body language into an affective state framework. PLoS ONE.

[17] Scharf B., Carroll J.A., Riley D.G., Chase C.C., Coleman S.W., Keisler D.H., Weaber R.L., Spiers D.E. (2010). Evaluation of physiological and blood serum differences in heat-tolerant (Romosinuano) and heat-susceptible (Angus) *Bos taurus* cattle during controlled heat challenge. J. Anim. Sci..

[18] Gaughan J.B. (2002). Respiration Rate and Rectal Temperature Responses of Feedlot Cattle in Dynamic, Thermally Challenging Environments. Ph.D. Thesis.

[19] Stewart M., Webster J., Verkerk G., Schaefer A., Colyn J., Stafford K. (2007). Non-invasive measurement of stress in dairy cows using infrared thermography. Physiol. Behav..

[20] Cook N., Schaefer A., Warren L., Burwash L., Anderson M., Baron V. (2001). Adrenocortical and metabolic responses to ACTH injection in horses: An assessment by salivary cortisol and infrared thermography of the eye. Can. J. Anim. Sci..

[21] Schaefer A., Cook N., Tessaro S., Deregt D., Desroches G., Dubeski P., Tong A., Godson D. (2004). Early detection and prediction of infection using infrared thermography. Can. J. Anim. Sci..

[22] Stewart M., Verkerk G., Stafford K., Schaefer A., Webster J. (2010). Non-invasive assessment of autonomic activity for evaluation of pain in calves, using surgical castration as a model. J. Dairy Sci..

[23] Brown-Brandl T.M., Nienaber J.A., Eigenberg R.A., Mader T.L., Morrow J., Dailey J. (2006). Comparison of heat tolerance of feedlot heifers of different breeds. Livest. Sci..

[24] Friard O., Gamba M. (2016). Boris: A free, versatile open-source event-logging software for video/audio coding and live observations. Methods Ecol. Evol..

[25] Uddin J., Phillips C.J.C., Goma A.A., McNeill D.M. (2019). Relationships between infrared temperature and laterality. Appl. Anim. Behav. Sci..

[26] Thom E.C. (1959). The discomfort index. Weatherwise.

[27] Benjamini Y., Hochberg Y. (1995). Controlling the false discovery rate: A practical and powerful approach to multiple testing. J. R. Stat. Soc. B Stat. Methodol..

[28] Glickman M.E., Rao S.R., Schultz M.R. (2014). False discovery rate control is a recommended alternative to Bonferroni-type adjustments in health studies. J. Clin. Epidemiol..

[29] Lees A.M. (2016). Biological Responses of Feedlot Cattle to Heat Load. Ph.D. Thesis.

[30] Young B.A., Hall A.B., Coombs B. (1993). Heat load in cattle in the Australian environment. Australian Beef.

[31] Shultz T. (1984). Weather and shade effects on cow corral activities. J. Dairy Sci..

[32] Mader T.L., Griffin D. (2015). Management of cattle exposed to adverse environmental conditions. Vet. Clin. N. Am. Food Anim..

[33] Neveux S., Weary D., Rushen J., Von Keyserlingk M., De Passillé A. (2006). Hoof discomfort changes how dairy cattle distribute their body weight. J. Dairy Sci..

[34] Chapinal N., De Passille A., Weary D., Von Keyserlingk M., Rushen J. (2009). Using gait score, walking speed, and lying behavior to detect hoof lesions in dairy cows. J. Dairy Sci..

[35] Navarro G., Santurtun E., Phillips C.J. (2017). Effects of simulated sea motion on stepping behaviour in sheep. Appl. Anim. Behav. Sci..

[36] Rogers L.J. (2008). Hand and paw preferences in relation to the lateralized brain. Philos. Trans. R. Soc. Lond. B Biol. Sci..

[37] Rogers L.J. (2010). Relevance of brain and behavioural lateralization to animal welfare. Appl. Anim. Behav. Sci..

[38] Austin N., Rogers L. (2007). Asymmetry of flight and escape turning responses in horses. Laterality.

[39] Phillips C.J.C. (2002). Cattle Behaviour and Welfare.

[40] Broom D.M., Fraser A.F. (2007). Domestic Animal Behaviour and Welfare.

[41] Proctor H.S., Carder G. (2014). Can ear postures reliably measure the positive emotional state of cows?. Appl. Anim. Behav. Sci..

[42] Boissy A., Aubert A., Désiré L., Greiveldinger L., Delval E., Veissier I. (2011). Cognitive sciences to relate ear postures to emotions in sheep. Anim. Welf..

[43] Gleerup K.B., Andersen P.H., Munksgaard L., Forkman B. (2015). Pain evaluation in dairy cattle. Appl. Anim. Behav. Sci..

[44] Smit T.H. (2002). The use of a quadruped as an in vivo model for the study of the spine–biomechanical considerations. Eur. Spine J..

[45] Hall W.B., McKeon G.M., Carter J.O., Day K.A., Howden S.M., Scanlan J.C., Burrows W.H. (1998). Climate change in Queensland’s grazing lands: II. An assessment of the impact on animal production from native pastures. Rangel. J..

[46] Johnson R.W. (2002). The concept of sickness behavior: A brief chronological account of four key discoveries. Vet. Immunol. Immunopathol..

[47] Lees A.M., Sejian V., Wallage A.L., Steel C.C., Mader T.L., Lees J.C., Gaughan J.B. (2019). The Impact of Heat Load on Cattle. Animals.

[48] Mandel R., Whay H.R., Nicol C.J., Klement E. (2013). The effect of food location, heat load, and intrusive medical procedures on brushing activity in dairy cows. J. Dairy Sci..

[49] Borderas T.F., de Passillé A.M., Rushen J. (2008). Behavior of dairy calves after a low dose of bacterial endotoxin. J. Anim. Sci..

